# Recent Progress in Anti-Obesity and Anti-Diabetes Effect of Berries

**DOI:** 10.3390/antiox5020013

**Published:** 2016-04-06

**Authors:** Takanori Tsuda

**Affiliations:** College of Bioscience and Biotechnology, Chubu University, Kasugai, Aichi 487-8501, Japan; tsudat@isc.chubu.ac.jp; Tel./Fax: +81-568-51-9659

**Keywords:** berries, anthocyanins, obesity, diabetes, degradation products, metabolites, bioavailability

## Abstract

Berries are rich in polyphenols such as anthocyanins. Various favorable functions of berries cannot be explained by their anti-oxidant properties, and thus, berries are now receiving great interest as food ingredients with “beyond antioxidant” functions. In this review, we discuss the potential health benefits of anthocyanin-rich berries, with a focus on prevention and treatment of obesity and diabetes. To better understand the physiological functionality of berries, the exact molecular mechanism of their anti-obesity and anti-diabetes effect should be clarified. Additionally, the relationship of metabolites and degradation products with health benefits derived from anthocyanins needs to be elucidated. The preventive effects of berries and anthocyanin-containing foods on the metabolic syndrome are not always supported by findings of interventional studies in humans, and thus further studies are necessary. Use of standardized diets and conditions by all research groups may address this problem. Berries are tasty foods that are easy to consume, and thus, investigating their health benefits is critical for health promotion and disease prevention.

## 1. Introduction

**Figure 1:**
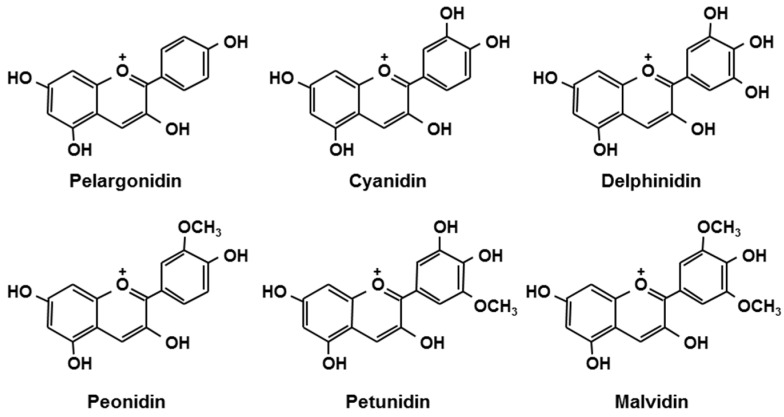
Chemical structures of anthocyanidins [15].

Berries are rich in polyphenols such as anthocyanins. Anthocyanins, belonging to the group flavonoids, are plant pigments that may appear red or purple, and are present in the form of glycosides [1,2]. Figure 1 shows the structures of the aglycone moieties of major anthocyanins [2]. The types and content of anthocyanins differ among varieties of berries, and depend on cultivation conditions and the timing of the harvest. The anthocyanin composition of some common berries is shown in Table 1 [3]. The components of berries are known for their strong anti-oxidant properties. It has been documented that anthocyanins have other health-related functions, such as improvement of visual and vascular function [4,5,6,7], anti-arteriosclerosis [8], anti-cancer [9,10], anti-obesity [11], anti-diabetes [12], and brain function-enhancing properties [13,14]. These favorable functions of berries cannot explained by their anti-oxidant properties alone, and thus, berries are now receiving great interest as food ingredients with “beyond antioxidant” functions. Also, the beneficial effects of berry component metabolites (e.g., anthocyanins) are becoming known.

Among a wide array of favorable functions of berries, their preventive and normalizing effect against obesity and diabetes will be discussed in this review, which will also outline findings regarding metabolism and absorption related to their anti-obesity and anti-diabetic effects. Furthermore, the latest interventional studies on the Metabolic Syndrome in humans are introduced. Finally, we summarize the future research needs regarding studies on berries’ favorable health benefits. 

## 2. Preventive and Normalizing Effect on Obesity and Diabetes

### 2.1. Anti-Obesity

The first report that demonstrated the preventive properties of anthocyanins against body fat accumulation was published by our group in 2003 [11]. Briefly, in C57BL/6J mice, a cyanidin 3-glucoside supplemented diet (C3G; 2 g/kg) significantly suppressed body fat accumulation induced by a high-fat diet (60% fat), and this was attributed to a reduction in lipid synthesis in the liver and white adipose tissue [11,16]. Anthocyanins act on adipose tissue, inducing changes, such as those in the expression levels of adipocytokines. We have reported that C3G or its aglycones induce upregulation of adiponectin, which enhances insulin sensitivity, in isolated rat and human adipocytes [17,18], but these events were not observed *in vivo*. Prior et al. reported that feeding a high-fat diet (45% fat) supplemented with anthocyanins extracted from blueberries significantly suppressed increases in body weight and body fat accumulation in C57BL/6 mice, while intake of lyophilized wild blueberry powder (WBP) did not demonstrate the same effect but induced body fat accumulation [19]. In a separate study, the same group reported that ingestion of blueberry juice did not significantly reduce the body weight gain and the weight of white adipose tissue (epididymal and retroperitoneal fat) in mice fed a high fat diet (45% of energy fat) [20]. A different group also reported similar findings [21]. On the other hand, Seymour et al. reported that supplementation of a high-fat diet (45% fat) with 2% WBP reduced the weight of intraperitoneal fat and increased the activity of the peroxisome proliferator-activated receptor (PPAR) in white adipose tissue and skeletal muscle in Zucker fatty rats [22]. Further, Vendrame *et al.* of the University of Maine reported that 8 weeks of feeding a diet supplemented with 8% WBP significantly increased blood adiponectin levels, and decreased levels of inflammation markers in white adipose tissue [23], and ameliorated dyslipidemia [24], but did not influence fasting blood glucose and insulin levels [25] in obese Zucker rats.

The effects of other types of berries have been tested. Ingestion of black raspberries did not significantly suppress body fat accumulation and weight gain in mice fed a high-fat diet (60% fat) [26,27,28]. On the other hand, ingestion of an aqueous extraction of mulberries suppressed increases in body weight [29]. Intake of tart cherry power significantly reduced body weight gain and the amount of retroperitoneal fat, suppressed upregulation of obesity-related inflammatory cytokines (IL-6 and TNF-α), and increased mRNA levels of PPARα and PPARγ in Zucker fatty rats [30]. In rats fed a high-fructose diet, intake of a chokeberry extract significantly suppressed increases in the weight of epididymal fat and blood glucose level, and at the same time, it significantly increased plasma adiponectin level and decreased plasma TNF-α and IL-6 levels [31]. Taken together, the anti-obesity effect of berries is controversial and findings are not consistent among studies. Use of standardized diets and conditions in all research groups may address this problem.

### 2.2. Anti-Diabetes

The author’s group reported that intake of purified anthocyanin (C3G) [16,32], bilberry anthocyanin extract (BBE) containing a variety of anthocyanins [12], and black soybean components (C3G and procyanidin) [33] significantly ameliorate hyperglycemia and insulin sensitivity in type 2 diabetic mice. It was reported by a different group that C3G and its metabolite protocatechuic acid caused activation of PPARγ, and also induced upregulation of Glut 4 and adiponectin in human adipocytes [34]. However, we demonstrated that C3G does not serve as a ligand of PPARγ, and did not observe C3G-induced upregulation of adiponectin *in vivo* [16,32]. Thus, it cannot be concluded that the effect of C3G against diabetes is due to activation of PPARγ-ligand or upregulation of adiponectin.

**Figure 2:**
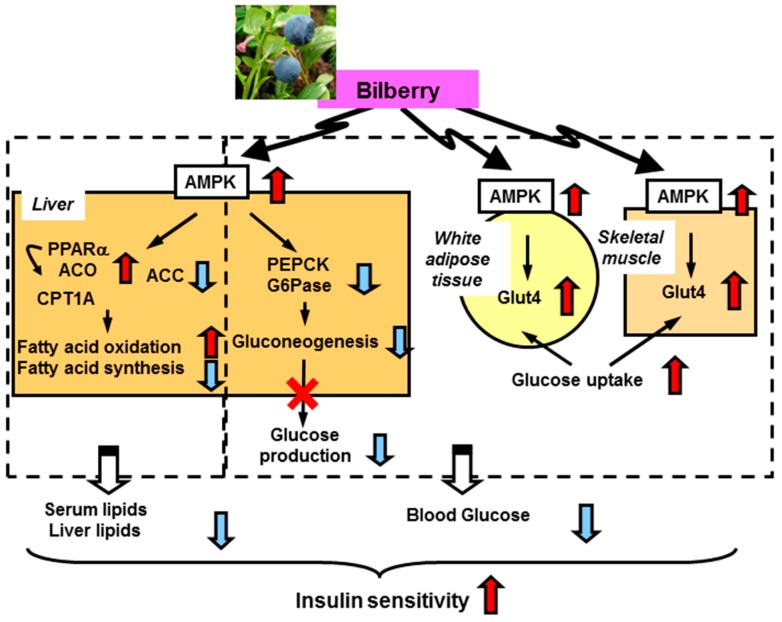
Proposed mechanism for amelioration of hyperglycemia and insulin sensitivity by dietary BBE [12]. ACC, acetylCoA carboxylase; ACO, acylCoA oxidase; CPT1A, carnitine palmitoyltransferase-1A; G6Pase, glucose-6-phosphatase; Glut4, glucose transporter 4; PEPCK, phosphoenol pyruvate carboxykinase.

BBE activates AMP-activated protein kinase (AMPK) in the white adipose tissue and skeletal muscle. This activation induces upregulation of glucose transporter 4 and enhancement of glucose uptake and utilization in these tissues. In the liver, dietary BBE suppresses gluconeogenesis (downregulation of glucose-6-phosphatase and phosphoenol pyruvate carboxykinase) via AMPK activation, which ameliorates hyperglycemia in type 2 diabetic mice. Meanwhile, in lipid metabolism, the AMPK activation induces phosphorylation of acetylCoA carboxylase and upregulation of PPARα, acylCoA oxidase , and carnitine palmitoyltransferase-1A gene expression in the liver. These changes lead to reductions in lipid content and enhance insulin sensitivity via reduction of lipotoxicity (Figure 2) [12].

Prevention of diabetes is an important task in the elderly and menopausal women, and ingestion of blueberries may be effective in its prevention. A research group at Louisiana State University reported that feeding a diet supplemented with 4% blueberry power for 12 weeks improved glucose intolerance and a fatty liver in post-menopausal mice [35].

**Figure 3:**
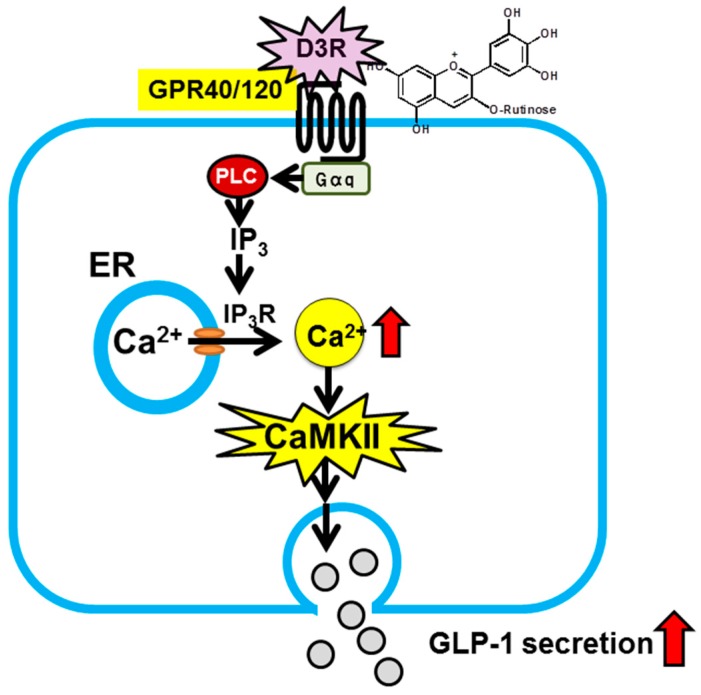
Proposed mechanism for stimulation of GLP-1 secretion by D3R in intestinal L-cells [15]. D3R activates G protein-coupled receptor (GPR), e.g., GPR40/120, on the L-cell surface. Activation induces IP3R-mediated release of intracellular Ca^2+^ from the endoplasmic reticulum. The elevation of cytosolic Ca^2+^ stimulates phosphorylation of Ca^2+^/calmodulin-dependent kinaseII (CaMKII). CaMKII activation leads to an increase in GLP-1 secretion from intestinal L-cells.

Regarding preventive and suppressive effects of anthocyanins against diabetes, our group recently discovered that anthocyanins induce secretion of glucagon-like peptide-1 (GLP-1), one of the incretins. “Incretins” is a general term for a group of peptide hormones that are secreted from the gastrointestinal tract upon food ingestion and act on pancreatic βcells, thereby inducing insulin secretion in a blood glucose concentration-dependent manner. There are two known incretins, one of which is GLP-1. Because enhancement of the action of GLP-1 is effective in prevention and treatment of type 2 diabetes, inhibitors of GLP-1 degradation and degradation-resistant GLP-1 receptor agonists are used for therapeutic purposes [36,37]. Use of food-derived factors to enhance secretion of endogenous GLP-1, thereby increasing blood GLP-1 levels, would serve as a new strategy [38]. There are several molecular species of anthocyanins. Their individual effects are still unknown, but their preventive and suppressive effect against diabetes may involve the action of GLP-1. Thus, we examined the inhibitory effect of each molecular species of anthocyanins on GLP-1 secretion, and as a result, discovered delphinidin 3-rutinoside (D3R) [15]. Furthermore, we documented that such secretion-inducing effect is mediated by G-protein coupled receptors (GPR40 or 120) and the downstream calcium-calmodulin dependent protein kinase II pathway (Figure 3) [15]. These findings indicate that anthocyanins, without being absorbed, can directly act within the intestine and exert health-related effects.

## 3. Health Benefits of Berries and Involvement of Their Metabolites

In 1999, we reported that protocatechuic acid is a metabolite of C3G [39]. Recently, phenolic acids (protocatechuic acid, syringic acid, vanillic acid, phloroglucinol aldehyde, phloroglucinol acid and gallic acid), which are degradation products or metabolites of anthocyanins, are of great interest in relation to health benefits of berries [40,41,42,43,44,45,46,47]. These phenolic acids were detected as metabolites in humans [48] 

It is believed that the bioavailability of anthocyanins is very low (about 0.1%). Also, their chemical structures indicate that they are prone to degradation, leading to the question why they exert favorable health benefits. A British group led by Kay reported a study that examined metabolism and absorption of ^13^C-labelled C3G in humans [49]. Briefly, 500 mg ^13^C-labelled C3G was ingested, and its excretion into the blood, urine, stool and expired air was monitored over a period of 48 h. It was shown that ^13^C was excreted even 24–48 h after ingestion and the detected conjugates and metabolites were diverse, and that the calculated bioavailability was ≥ 12.38% ± 1.38%. In addition to conjugates of C3G and its aglycone (cyanidin), the following degradation products and metabolites were detected in the same study: protocatechuic acid and its glucuronate conjugate or sulfate conjugate; vanillic acid and its derivatives and conjugates; phenylacetic acids (3,4-dihydroxyphenyl acetic acid and 4-hydroxyphenylacetic acid; phenylpropenoic acids (caffeic acid and ferulic acid); and hippuric acid. It is likely that C3G undergoes chemical degradation in a considerably complicated metabolic processes, influenced by enteric bacteria, and is re-absorbed. The same group prepared various C3G metabolites, and reported that these metabolites at physiological concentrations suppress inflammation in human vascular endothelial cells [50,51].

A British group led by Spencer also reported that a wide range of phenolic acids in plasma, most likely to be degradation products and metabolites of anthocyanins, were present after ingestion of a blueberry drink [52]. Some of these compounds were detected in blood and peaked only 1 h after ingestion, while others were detected several hours after ingestion.

A Canadian group led by Kalt examined anthocyanin metabolites after ingestion of 250 mL blueberry juice in humans, and found that metabolites were excreted into urine over a period of 5 days [53]. This suggests that these metabolites circulate within the enterohepatic loop, and remain in the body for a long period. To confirm this, a tracer study conducted by a British group [49] can provide us with some important information. Concretely, examination of metabolism and absorption of various ^13^C-labelled types of anthocyanin species may help confirm circulation of the metabolites within the enterohepatic loop.

A study by a group at North Carolina State University investigated whether degradation of anthocyanins by enteric bacteria and resulting products, namely phenol acids, are responsible for the health benefit of anthocyanins. It was shown that feeding mice a diet containing 1% black currant powdered extract (32% anthocyanins) for 8 weeks suppressed weight gain and improved glucose metabolism. However, these effects were observed in mice with normal gut microbiome, but not in mice with intestinal flora altered by antibiotics [54]. It is of great interest that the study showed that metabolites generated by enteric bacteria from berry components are involved in exerting health-related effects.

**Figure 4:**
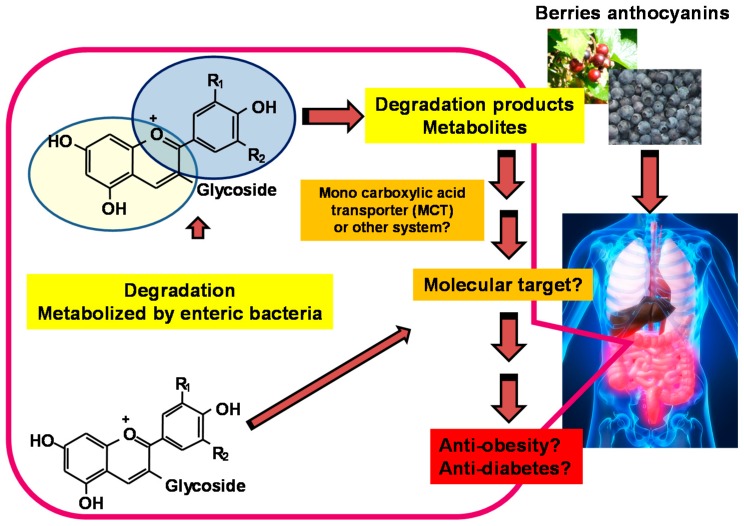
Degradation products or metabolites derived from berry anthocyanins can have an impact on health.

Taken together, the link between functional doses of anthocyanin or berry intake and metabolite concentrations may be explored to evaluate the health benefits of berries (Figure 4).

## 4. Interventional Studies in Humans

In this section, recent intervention studies of berries in humans are introduced. Obesity and diabetes are closely linked to cardiovascular disease. Therefore, some studies shown here include the role of berries in cardiovascular disease prevention. 

Basu *et al*., conducted a randomized study wherein 48 obese men and women (mean body mass index (BMI), 37.8) ingested lyophilized blueberries containing anthocyanins (742 mg) for 8 weeks. It was shown that blood pressure was significantly improved and oxidized low-density lipoprotein (LDL)-cholesterol levels were decreased, while the blood glucose levels, body weights and waist circumferences were not improved in the group who ingested blueberries [55]. On the other hand, a US research group conducted a randomized double-blind comparison wherein improvement of insulin sensitivity was demonstrated in 32 men and women who ingested blueberry powder (669 mg/day anthocyanins) for 6 weeks [56]. Furthermore, a research team involving US, British and Singaporean groups jointly conducted an epidemiological study, and reported significant decreases in the risk of type 2 diabetes in the group that ingested a large amount of anthocyanins [57]. A British group also reported the interrelation between anthocyanin intake and decreases in levels of blood insulin and inflammatory markers [58]. A US group reported that high anthocyanin intake significantly reduced the risk of myocardial infarction in 93,600 young and middle-aged women [59]. On the other hand, ingestion of elderberries (500 mg/day, 12 weeks) did not improve the profile of cardiovascular disease markers in 52 post-menopausal women [60]. Also, a randomized double-blind comparison found that ingestion of purple carrots (daily intake of anthocyanins and phenolic acids, 118.5 mg and 259.2 mg, respectively) for 4 weeks did not influence the body weight, LDL-cholesterol level and blood pressure, but it significantly reduced the high-density lipoprotein (HDL)-cholesterol level in 16 obese men (mean BMI, 32.8) [61]. In summary, the preventive effects of berries and anthocyanin-containing foods on the Metabolic Syndrome are currently not always supported by findings of interventional studies in humans, and thus further studies are necessary.

## 5. Future Research Needs and Prospects

Finally, we present the research needs on the beneficial effects of berries and anthocyanins.

First, in studies on the suppressive/normalizing effects of anthocyanins on obesity and diabetes, interrelation between chemical structures of anthocyanin molecules and their various beneficial effects remain largely unclear. On the other hand, simultaneous consumption of various types of anthocyanins may be more beneficial in some cases. Thus, it is crucial to elucidate which type of anthocyanin species or compositions of the mixture are most beneficial for their health benefits.

Second, the relationships of metabolites and degradation products derived from anthocyanins with health benefits need to be elucidated. The following questions should be answered: whether health-related effects of berries can be explained solely by metabolites and degradation products of anthocyanins; what types and quantities of metabolites and degradation products of anthocyanins are necessary for them to exert their beneficial effects; whether differences in intestinal flora influence the beneficial effects of berries; and conversely, whether ingestion of berries affects intestinal flora.

Third, it is necessary to determine whether the effects caused by anthocyanins solely explain the health benefits of berries, or the co-presence of other berry components is essential for their beneficial effects.

Lastly, health benefits that may result from berries have not yet been fully studied in humans, and findings are not consistent among studies. Use of standardized diets and conditions in all research groups may address this problem.

Berries are tasty foods that are easy to consume, and thus, investigating their health benefits is critical for health promotion and disease prevention. 

## Figures and Tables

**Table 1:** Anthocyanin Content in Fresh Berries (Reprinted with permission from [3]. Copyright 2008 American Chemical Society).

Berries	Anthocyanins	mg/g Extract ^a^
Bilberry	cyanidin 3-galactoside	3.70
	cyanidin 3-glucoside	4.05
	cyanidin 3-arabinoside	2.54
	delphinidin 3-galactoside	4.58
	delphinidin 3-glucoside	4.73
	delphinidin 3-arabinoside	3.53
	peonidin 3-galactoside	0.46
	petunidin 3-halactoside	1.52
	petunidin 3-glucoside	2.94
	petunidin 3-arabinoside	0.84
	malvidin 3-arabinoside	0.81
	peonidin 3-glucoside/malvidin 3-galactoside	3.48
	peonidin 3-arabinoside/malvidin 3-glucoside	3.62
Blackberry	cyanidin 3-glucoside	7.17
	cyanidin 3-rutinoside	0.06
	cyanidin 3-arabinoside	0.05
	cyanidin 3-xyloside	0.47
	cyanidin 3-(6-malonoyl)glucoside	0.3
	cyanidin 3-dioxaloylglucoside	2.05
Blackcurrant	cyanidin 3-glucoside	1.1
	cyanidin 3-rutinoside	7.08
	delphinidin 3-glucoside	2.94
	delphinidin 3-rutinoside	9.79
	peonidin 3-rutinoside	0.11
	petunidin 3-rutinoside	0.18
Blueberry	cyanidin 3-galactoside	0.28
	cyanidin 3-glucoside	0.04
	cyanidin 3-arabinoside	0.12
	delphinidin 3-galactoside	1.37
	delphinisin 3-glucoside	0.13
	delphinidin 3-arabinoside	0.74
	peonidin 3-galactoside	0.15
	petunidin 3-galactoside	1.07
	petunidin 3-glucoside	0.11
	petunidin 3-arabinoside	0.46
	marlidin 3-arabinoside	1.75
	peonidin 3-glucoside/malvidin 3-galactoside	3.65
	peonidin 3-arabinoside/malvidin 3-glucoside	0.43
Strawberry	cyanidin 3-glucoside	0.09
	pelargonidin 3-glucoside	5.07

^a^ Values are expressed as mean of triplicate analyses for each sample.
